# Anti-VEGF treatment for macular conditions

**Published:** 2025-01-31

**Authors:** Michael Mikhail

**Affiliations:** 1 Consultant Ophthalmologist and Vitreoretinal Surgeon: Kabgayi Eye Unit, Muhanga, Rwanda and Senior Lecturer: College of Medicine and Pharmacy, University of Rwanda.

**The introduction of anti-VEGF drugs has revolutionised the management of macular conditions that were previously untreatable.**

**Figure:**
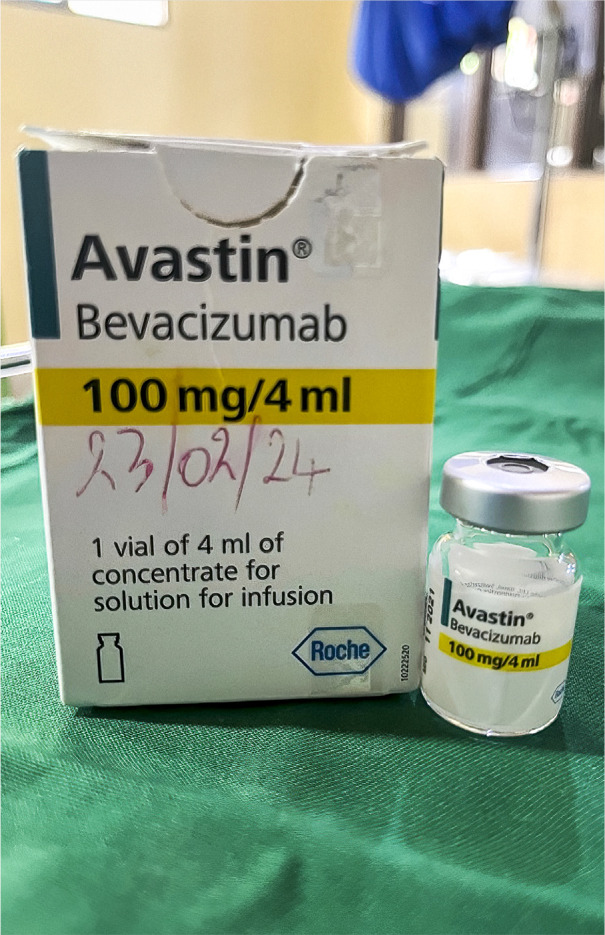
Many eye units still rely on bevacizumab (Avastin) due to its lower cost.

© MICHAEL MIKHAIL CC BY-NC-SA 4.0

VEGF stands for vascular endothelial growth factor. It is a protein that plays a critical role in the growth and development of new blood vessels. VEGF production is increased by a lack of oxygen. Once tissues (e.g. in the retina) sense the lack of oxygen, more and more VEGF is produced, leading to growth of new blood vessels in an attempt to provide more oxygen to the tissues. However, VEGF also increases the permeability of fine blood vessels (capillaries), so these new blood vessels tend to be abnormal and bleed or leak fluid and proteins into the surrounding tissue. In diseases of the macula, like age-related macular degeneration and diabetic macular oedema, VEGF is often overproduced, leading to retinal damage and several blinding complications.

The introduction of anti-VEGF drugs given as intravitreal injections, has revolutionised the treatment of these previously untreatable conditions. Many patients can now achieve a reasonable visual acuity, especially with early diagnosis and timely treatment. The most commonly used anti-VEGF drugs are bevacizumab (Avastin), ranibizumab (Lucentis), and aflibercept (Eylea). These three medications have been in use for more than a decade, with proven safety and efficacy in treating age-related macular degeneration and diabetic macular oedema.

## Limitations and challenges

Because anti-VEGF drugs do not cure the underlying pathology, patients need repeated injections and regular monitoring, and their treatment may have to be continued indefinitely.

In low-income settings, affording anti-VEGF treatment remains a challenge. The cost of anti-VEGF drugs is still relatively high, and many eye units rely on bevacizumab (Avastin) due to its lower cost, making it more affordable for our patients, with comparable outcomes to other anti-VEGF drugs.^1^ However, more frequent administration is required, and the cost of multiple clinic visits means that many people cannot afford ongoing treatment.

In high-income settings, cost is less of an issue. However, many eye units are struggling to keep up with the demands of providing multiple injections and regular review for the growing number of patients benefiting from anti-VEGF treatment. This has prompted the search for new anti-VEGF drugs that can reduce the number of clinic visits each patient needs. Two new drugs that can increase treatment intervals to 12 or even 16 weeks are faricimab (Vabysmo 6 mg), and an increased 8 mg dose of aflibercept (Eylea). Other advances are underway and yet to be licensed. One of these is the port delivery system (PDS) by Roche: an implant surgically placed through the ciliary body which acts as a reservoir of ranibizumab that is refilled every 6 months. Several companies are working on administering relevant drugs as eye drops; this would either avoid the need for injections or lengthen the interval between them.

Anti-VEGF biosimilars are highly similar versions of ‘originator’ drugs like bevacizumab (Avastin) and ranibizumab (Lucentis). Biosimilars are designed to provide the same safety, quality, and efficacy but at a lower cost, making them ideal for resource-limited settings. However, to the best of my knowledge, they are not yet available in most African countries, including Rwanda. Their introduction could significantly improve access and affordability of retinal care across the continent.

**Treatment regimen for established anti-VEGF drugs**

Anti-VEGF drugs need to be given in the long term for best results. All of them require a loading dose of 3–4 injections, spaced 4 weeks apart. After the loading dose, review patients and discuss a further treatment regimen (this is usually one injection every 8 weeks for aflibercept and faricimab).

**Note:** The dose and frequency of administration is the same for diabetic macular oedema and neovascular age-related macular degeneration.
